# Bovine Viral Diarrhea Virus (BVDV) in White-Tailed Deer (*Odocoileus virginianus*)

**DOI:** 10.3389/fmicb.2016.00945

**Published:** 2016-06-20

**Authors:** Thomas Passler, Stephen S. Ditchkoff, Paul H. Walz

**Affiliations:** ^1^Department of Clinical Sciences, College of Veterinary Medicine, Auburn UniversityAuburn, AL, USA; ^2^School of Forestry and Wildlife Sciences, Auburn UniversityAuburn, AL, USA; ^3^Department of Pathobiology, College of Veterinary Medicine, Auburn UniversityAuburn, AL, USA

**Keywords:** bovine viral diarrhea virus, interspecific transmission, *Odocoileus virginianus*, wildlife reservoir, white-tailed deer

## Abstract

Bovine viral diarrhea virus (BVDV) is the prototypic member of the genus *Pestivirus* in the family *Flaviviridae*. Infections with BVDV cause substantial economic losses to the cattle industries, prompting various organized control programs in several countries. In North America, these control programs are focused on the identification and removal of persistently infected (PI) cattle, enhancement of BVDV-specific immunity through vaccination, and the implementation of biosecure farming practices. To be successful, control measures must be based on complete knowledge of the epidemiology of BVDV, including the recognition of other potential sources of the virus. BVDV does not possess strict host-specificity, and infections of over 50 species in the mammalian order Artiodactyla have been reported. Over 50 years ago, serologic surveys first suggested the susceptibility of white-tailed deer (*Odocoileus virginianus*), the most abundant free-ranging ruminant in North America, to BVDV. However, susceptibility of white-tailed deer to BVDV infection does not alone imply a role in the epidemiology of the virus. To be a potential wildlife reservoir, white-tailed deer must: (1) be susceptible to BVDV, (2) shed BVDV, (3) maintain BVDV in the population, and (4) have sufficient contact with cattle that allow spillback infections. Based on the current literature, this review discusses the potential of white-tailed deer to be a reservoir for BVDV.

## Introduction

Since the first descriptions of bovine viral diarrhea virus (BVDV) in North American cattle in 1946 (Childs, 1946; Olafson et al., 1946), great strides have been made in understanding the virological, epidemiological, and pathophysiological features that have allowed BVDV to become one of the most important viral pathogens of cattle worldwide. The elucidation of the pathophysiology of persistently infected (PI) cattle and recognition of PI animals as the most important source for direct and indirect transmission of BVDV (McClurkin et al., 1984; Brownlie et al., 1987) has shaped current BVDV control measures to focus on eradication of PI cattle and prevention of *in utero* infections through vaccination and biosecurity measures. The development of molecular diagnostic techniques has allowed the classification of pestiviruses by genotypic diversity rather than by the mammalian host from which a virus was isolated and emphasized that pestiviruses lack strict host specificity (Nettleton, 1990; Giangaspero and Harasawa, 2007). Interestingly, reports of apparent BVDV-associated disease outbreaks in heterologous hosts were published within a few years following the first description of BVDV in cattle (Richards et al., 1956; Brass et al., 1966). While the involvement of BVDV is uncertain or unlikely in some early reports of apparent heterologous infections, numerous studies have since demonstrated that BVDV infections are possible in many species of the mammalian order Artiodactyla, including domestic small ruminants, buffalo, swine, Old and New World camelids, and free-ranging and captive wildlife (Passler and Walz, 2010). The implications of heterologous BVDV infections including adverse effects on health and reproduction of affected species, ability to maintain BVDV in the population, and potential to become a reservoir host are still incompletely understood. To be a reservoir for BVDV and impede eradication efforts, a heterologous host species has to be: (1) susceptible to infection, (2) able to shed BVDV, (3) maintain the virus within individual hosts or the host-population, and (4) have sufficient contact with susceptible cattle herds. This review summarizes the current literature on BVDV infection in white-tailed deer and discusses whether this species has the potential to be a reservoir for BVDV.

## Susceptibility of White-Tailed Deer to Infection with BVDV

### Serologic Evidence of Susceptibility

First evidence of susceptibility of white-tailed deer to BVDV infection was documented by Kahrs et al. (1964), who examined 200 sera from New York for presence of BVDV antibodies and detected a seroprevalence rate of 3% (Kahrs et al., 1964). Since then, other North American groups have investigated the presence of antibodies against BVDV in white-tailed deer and generally detected low seroprevalence rates (**Table 1**). Reported BVDV seroprevalence rates in white-tailed deer tend to be lower than those reported for other cervids such as mule deer (Stauber et al., 1977; Couvillion et al., 1980; Aguirre et al., 1995; Van Campen et al., 2001; Roug et al., 2012; Myers et al., 2015). Whether this difference reflects greater rates of contact between mule deer and cattle, or maintenance of BVDV in mule deer and transmission among conspecifics, is currently unknown. However, there are key differences in white-tailed and mule deer life history patterns that could explain these trends. Many mule deer populations exhibit migratory behavior, where they move elevationally between summer and winter ranges (Nicholson et al., 1997). Although most studies indicate that mule deer tend to avoid cattle (Stewart et al., 2002; Dohna et al., 2014), migratory movements could increase contact rates between mule deer and cattle due to direct contact around feeding sources, or indirect contact via use of resources that are partitioned temporally, particularly during periods when resources are limiting (Stewart et al., 2002). While white-tailed deer populations in northern regions with severe winter climates also migrate between summer and winter ranges (Nelson, 1998), cattle in these regions are generally confined to conventional production systems that include fenced enclosures and indoor containment facilities (Dohna et al., 2014), thus causing contact rates between wild ungulates and cattle to be less in these settings. Differences in vegetation patterns of cattle grazing lands across North America may also contribute to disparity between BVDV prevalence rates in mule and white-tailed deer. Whereas much cattle grazing east of the Rocky Mountains (excluding some areas such as southern Texas) is dominated by pastures of exotic or native grasses with minimal structural and vegetative diversity, much of the grazing land in western North America is on rangelands and has a greater preponderance of preferred deer browse species (Rickard et al., 1975; Loeser et al., 2007; Wagoner et al., 2013), thereby increasing the probability of contact between deer and cattle. Considering the ranges of white-tailed deer and mule deer, the overlap in dietary items on native range, and limited availability of water resources, it readily becomes apparent that this could be a contributing factor.

**Table 1:** **Reported seroprevalence rates in free-ranging white-tailed deer**.

Location	Seroprevalence rate	Reference
New York State	3%	Kahrs et al., 1964
New York State, two locations	5.7 and 7.0%	Friend and Halterman, 1967
Maryland and Virginia, one wildlife refuge each	0/5 and 2/5 deer	Davidson and Crow, 1983
Florida, one location	0/6	Davidson et al., 1987
Quebec, one location	0%	Sadi et al., 1991
Colorado, one location	1/5 deer	Creekmore et al., 1999
Southern Minnesota, nine locations	25% (southeast) and 41% (southwest)	Wolf et al., 2008
Northeastern Mexico, 15 locations	63.5%	Cantu et al., 2008
Alabama, 23 locations	1.2%	Passler et al., 2008
Central New York State and four locations in Pennsylvania	6.01 and 0.34%, respectively	Kirchgessner et al., 2012
New York State	7.48%	Kirchgessner et al., 2013


Two recent studies documented relatively high rates of BVDV antibody presence in white-tailed deer (Cantu et al., 2008; Wolf et al., 2008). A serosurvey conducted on 15 ranches in Northeastern Mexico, in which the overall rate of seropositive white-tailed deer was 63.5%, demonstrated that significantly greater seroprevalence rates were present on ranches where cattle were present, as compared to ranches without cattle. Other factors that were associated with increased prevalence rates of BVDV antibodies included the abundance of brush and exotic grasses, continuous grazing practices, and lower deer density (Cantu et al., 2008). In a study performed in Minnesota, a greater percentage (46%) of deer were seropositive in the southwestern study area that contained lower cattle densities, mostly composed of beef cattle herds. While the southeastern study area (seroprevalence in white-tailed deer: 25%) had greater cattle densities, the majority of these herds were composed of dairy cattle. The authors suggested that greater opportunities for contact between white-tailed deer and cattle exist on beef operations, where cattle are kept on pastures, rather than with dairy cattle that are largely confined (Wolf et al., 2008). A recent study documented that seroprevalence rates in New York (6.01%) were greater than those in Pennsylvania (0.34%), but beef cattle densities were similar in both sampling areas. However, the dairy cattle and total cow/calf densities were significantly greater in New York which may have contributed to increased rates of BVDV infection in white-tailed deer (Kirchgessner et al., 2012). Contact with cattle is the likely source for BVDV infections of white-tailed deer, as is further suggested by the absence of BVDV antibodies in a population of deer that had no direct or indirect contact with cattle in over 50 years (Sadi et al., 1991). However, maintenance of BVDV within deer populations independent of contact with cattle may also be possible, especially if PI deer are present.

### Experimental Infection of White-Tailed Deer with BVDV

Susceptibility of white-tailed deer to infection with BVDV was first confirmed experimentally by Van Campen et al. (1997), who intranasally inoculated four mule deer fawns and one white-tailed deer fawn at 5–6 months of age with BVDV NY-1. In that study, infection with BVDV did not result in noticeable clinical signs or changes of white blood cell counts. However, of the five fawns, only one mule deer was seronegative to BVDV at the time of inoculation, which may have subdued the expression of clinical signs, but did not prevent viremia and seroconversion. Shedding of BVDV was demonstrated on nasal swab samples from three mule deer fawns, but not the white-tailed deer (Van Campen et al., 1997). In another study, BVDV-naïve white-tailed deer fawns were inoculated with BVDV 1b RO3-24272 or BVDV 2 RO3-20663 isolated from white-tailed deer carcasses in South Dakota (Ridpath et al., 2007b). All fawns became infected as indicated by seroconversion and/or viremia, and clinical signs including pyrexia, lethargy, and coughing were observed. On days 3 and 6 of the study, pronounced lymphopenia was observed in inoculated animals, and circulating lymphocyte counts were reduced by 50 and 60% in fawns inoculated with BVDV 1b and BVDV 2, respectively (Ridpath et al., 2007b). On day 3, following infection of these fawns, some leukocyte subpopulations were almost completely depleted but had recovered by day 9 of the study (Mark et al., 2005, 2006). Lymphoid depletion, apoptosis, and lymphoid necrosis were also detected in lymphoid tissues of four fawns inoculated with BVDV 1 544 WTD from a free-ranging white-tailed deer in Indiana (Raizman et al., 2009). Following inoculation, these fawns did not have clinical signs of BVDV-associated disease, but all were positive by virus isolation on tissues (Raizman et al., 2009). In a follow-up study using BVDV 1 544 WTD, infection of five white-tailed deer fawns similarly resulted in absence of clinical signs despite evidence of infection in all fawns, presence of viral RNA in buffy coat, nasal swab, and rectal swab samples, and marked lymphoid atrophy in the Peyer’s patches (Negron et al., 2012).

**FIGURE 1:**
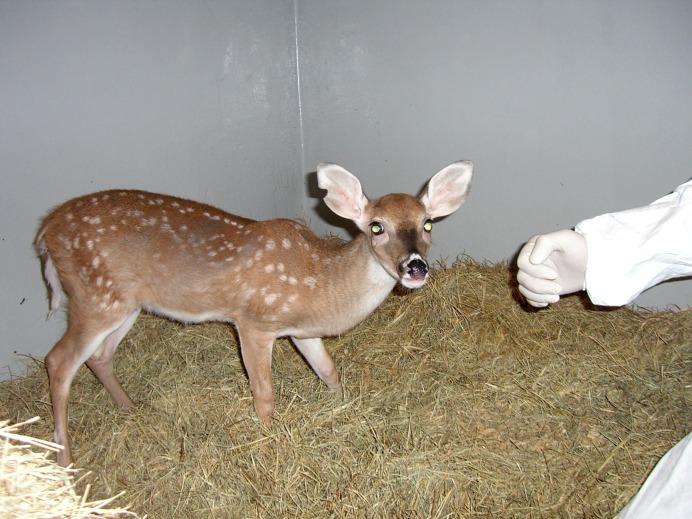
**Persistently infected fawn at approximately 4 months of age**. The fawn was hand-raised in an isolation room and remained free from clinical signs of disease until sudden death at 5 months of age.

The most severe clinical effects of BVDV in white-tailed deer may result from infection during pregnancy. Using BVDV 1b RO3-24272 or BVDV 2 RO3-20663 of white-tailed deer origin, Ridpath et al. (2008) investigated the outcome of infection of pregnant white-tailed deer (eight seronegative and two seropositive) that were inoculated in the first trimester at 6–7 weeks of gestation (Ridpath et al., 2008). BVDV infection resulted in clinical signs including depression, ill-thrift, and drooling within 7 days of inoculation. Four of the 10 inoculated deer died, and only three does gave birth to live fawns. The remaining pregnancies resulted in abortion, fetal resorption, and fetal mummification with evidence of transplacental BVDV infection in some fetal tissues. Three apparently healthy liveborn fawns were born to does seropositive at the time of inoculation, and these fawns were free from BVDV-infection and seronegative at birth. Two additional fawns were born by a seronegative doe. These fawns were determined to be seronegative at birth and positive for BVDV on virus isolation in buffy coat samples and antigen detection in skin samples (Ridpath et al., 2008). While the death of the fawns within 24 h of their birth prevented further confirmation of their persistent infection, the successful isolation of BVDV 163 days after inoculation strongly supports their PI status. In another study, nine pregnant does were inoculated with BVDV 1 BJ and BVDV PA131 at approximately 50 days of gestation (Passler et al., 2007). While clinical signs of BVDV infection were not observed during examinations from a distance, pregnancy losses occurred in 8/9 does. Whether these losses were BVDV-associated or caused by immobilization procedures during BVDV inoculation is uncertain; however, one of the fetuses of the doe that carried the pregnancy to term was delivered mummified, suggesting BVDV-associated reproductive failure. The fetal mummy was a twin to a liveborn, viable fawn that was hand-raised in an isolation facility. This fawn was confirmed to be PI with BVDV 2 PA131 based on virus isolation of serum, buffy coat, and nasal swab samples; RT-PCR of the serum and buffy coat; and detection of BVDV antigen in an ear notch sample by immunohistochemistry (Passler et al., 2007). The PI fawn remained free from clinical signs of disease and developed normally until it died suddenly at 5 months of age (**Figure 1**).

In contrast to the severe reproductive losses encountered in the above mentioned studies, the pregnancy of all does infected with BVDV by exposure to PI animals in two other studies advanced to term (Passler et al., 2009, 2010). In both studies, successful infection of all does was confirmed by demonstration of seroconversion, but with exception of two stillborn twins, all fawns were liveborn. Whether the observed differences in gestational viability in these studies were due to differences in the viral isolates used for inoculation or differences between the routes of exposure (intranasal inoculation vs. cohabitation with PI) is currently unknown. Previous studies in cattle (Brock and Cortese, 2001; Bielefeldt-Ohmann et al., 2008) and white-tailed deer (Passler et al., 2007) suggested that BVDV 2 isolates are able to cause transplacental infections more readily, and in pregnant goats, pregnancy losses were much more frequently associated with BVDV 2 PA131 than with BVDV 1b AU526 (Passler et al., 2014). Inoculation of pregnant deer during later gestation with BVDV 2 RO3-20663 resulted in pregnancy losses in 3/5 does inoculated during the second trimester and birth of apparently healthy, seropositive fawns from does infected in the third trimester, confirming that BVDV infections of pregnant white-tailed deer are very similar to those of pregnant cattle (Ridpath et al., 2012).

Similar to the outcome of acute BVDV infection of white-tailed deer, the BVDV antigen distribution in PI white-tailed deer was recently demonstrated to be largely equivalent to that of PI cattle (Duncan et al., 2008a; Passler et al., 2012b). As in PI cattle, BVDV antigen was distributed broadly in many organ systems with greatest antigen staining in epithelial tissues. Skin samples were demonstrated to be a suitable sample for BVDV antigen detection in white-tailed deer. However, in lymphatic and alimentary tissues, which are commonly collected for BVDV diagnosis in cattle, BVDV antigen was detected at lower frequency and intensity, which may in part be due to moderate to severe lymphoid depletion in tissues of PI white-tailed deer (Duncan et al., 2008a; Passler et al., 2012b). Therefore, diagnosis of BVDV infections in white-tailed deer should not rely solely on lymphatic and alimentary tissues, but include samples from the hepatobiliary, integumentary, neurologic, and reproductive organs, which were demonstrated to contain the most pronounced BVDV antigen (Passler et al., 2012b).

### Detection of BVDV in Free-Ranging White-Tailed Deer

The first isolation of BVDV from free-ranging white-tailed deer was made from two animals that were gunshot due to illness in two adjacent counties in South Dakota (Chase et al., 2004, 2008). BVDV was detected in multiple tissues of both deer by virus isolation and immunohistochemistry, and the BVDV antigen distribution in ear skin from both animals was consistent with the distribution in PI cattle. The authors also reported that following detection of the two positive deer, approximately 600 samples collected from white-tailed deer, elk, and mule deer in South Dakota were screened by immunohistochemistry, but all were negative for BVDV antigen (Chase et al., 2004, 2008). Similarly, in a recent study, ear notches from 367 hunter-harvested white-tailed deer were evaluated by antigen-capture ELISA, and BVDV antigen was not detected (Ilha et al., 2012). Three other surveys utilized immunohistochemistry or ELISA techniques to investigate the occurrence of BVDV in free-ranging cervids in the US. In Alabama, 1 of 406 ear notches (0.2%; 95% CI: 0–0.6%) was positive by immunohistochemistry, and the antigen distribution resembled that of PI cattle (Passler et al., 2008). A survey that screened 5597 deer (including 141 white-tailed deer) for BVDV by immunohistochemistry, detected BVDV antigen in one mule deer from which BVDV 1 was subsequently isolated, but BVDV antigen was not detected in the white-tailed deer (Duncan et al., 2008b). The overall apparent prevalence for BVDV-infected deer in Colorado was 0.03% (95% CI: 0–0.1%; Duncan et al., 2008b). In Indiana, 2 of 745 (0.26%, 95% CI: 0.1–0.64) white-tailed deer were positive for BVDV by antigen capture ELISA, and subsequently a cytopathic and a non-cytopathic BVDV were isolated (Pogranichniy et al., 2008). During BVDV testing in cattle herds, acutely infected animals may occasionally cause positive results; however, skin biopsy (ear notch) testing by immunohistochemistry or antigen capture ELISA is considered to be specific for detection of PI c (Walz et al., 2010). To date, antigen detection assays have not been validated for use in white-tailed deer, but positive samples are assumed to have been collected from PI animals. In several experimental infection studies, the BVDV antigen distribution in ear notches of PI white-tailed deer as detected by immunohistochemistry was consistent with that of PI cattle (Passler et al., 2007, 2009, 2010; Ridpath et al., 2008); however, samples from acutely infected deer or deer infected with other pestiviruses have not been evaluated.

Surveys using samples from hunter-harvested deer potentially underestimate the true prevalence of PI animals as they contain an inherent bias regarding the classes of animals sampled. Deer harvests greatly underrepresent young of the year due to selectivity of hunters for adults and their “trophy” status (Ditchkoff et al., 2000), thus significantly reducing the probability of hunters harvesting PI animals, which may die early in life due to complications from BVDV infection. Additionally, deer that manifest symptoms associated with PI status may be less desirable for harvest due to previously reported occurrence of poor body condition, ill-thrift, and smaller body size (Chase et al., 2008). Furthermore, studies that screen for disease pathogens in wildlife often utilize simple random sampling methods that survey multiple, widespread populations across larger regions, as was performed in most studies that evaluated BVDV in white-tailed deer (Kahrs et al., 1964; Pogranichniy et al., 2007; Duncan et al., 2008b; Passler et al., 2008). These surveys may not adequately acknowledge social structures of deer populations and therefore miss evidence of BVDV hotspots as a result of intrapopulational maintenance. White-tailed deer exist in matrilineal groups in which female deer disperse only over small distances according to the rose petal hypothesis (Porter et al., 1991). Cantu et al. (2008) demonstrated that the overall prevalence of BVDV antibodies in captured white-tailed deer was 63.5%; however, large variations among the 15 different ranches were detected, and while the seroprevalence was as low as 11% on one farm, 100% of sampled deer were seropositive on another. The potential impact of biased data associated with the inclusion of specifically targeted animals should be considered during surveillance studies for BVDV. For example, surveillance programs for chronic wasting disease in white-tailed deer commonly include targeting animals that are “symptomatic” (Evans et al., 2014). While this approach may be beneficial for presence/absence surveillance or identification of “hot spots,” inclusion of these data in prevalence studies could artificially inflate prevalence rates. These issues suggest that care should be taken when designing surveys for BVDV and other diseases that may manifest themselves in more clumped distributions (Nusser et al., 2008).

## Shedding and Transmission of BVDV by White-Tailed Deer

In cattle, BVDV is shed in most excretions and secretions, including nasal discharge, saliva, tears, milk, urine, feces, and semen (Houe, 1995). While studies investigating the possible routes of BVDV transmission from infected white-tailed deer are sparse, the broad distribution of BVDV described in tissues of PI deer (Duncan et al., 2008a; Passler et al., 2012b) suggests that excretion of virus may be similar to cattle, and shedding was demonstrated following experimental acute infections and in PI white-tailed deer (Passler et al., 2007, 2009; Raizman et al., 2009; Ridpath et al., 2009; Negron et al., 2012). In a study using BVDV 1a 544 WTD for experimental infection of four seronegative fawns at approximately 3 weeks of age, BVDV was demonstrated on the nasal swab samples of two fawns and the rectal swab sample of one fawn by RT-PCR 7 days after infection. In contrast, BVDV was not detected in samples from the other two fawns on days 7 or 14 of the study (Raizman et al., 2009). Following inoculation of five seronegative fawns with BVDV 1a 544 WTD, BVDV RNA was detected in nasal, oral, and rectal swab samples of five, four, and five fawns, respectively, as early as 3 days after inoculation and for up to 18 days (Negron et al., 2012). Two days after inoculation, each fawn was cohabitated with 1–2 seronegative calves in an isolation room for 19 days. Direct contact with the infected fawns resulted in BVDV infection in four of six calves (Negron et al., 2012), demonstrating that acutely infected white-tailed deer can shed sufficient amounts of BVDV to transmit the virus to cattle that are in close contact. Another study investigated the potential for BVDV transmission from acutely infected white-tailed deer to seronegative calves by indirect contact (Ridpath et al., 2009). Fawns were inoculated with BVDV 2 RO3-20663 of white-tailed deer origin in isolation rooms that shared circulating air with rooms containing seronegative calves. To simulate opportunities of indirect contact between species, fawns and calves were bottle-fed using shared nipple bottles, and every second day, without prior cleaning, the calves were rotated into rooms that had been previously occupied by fawns. While BVDV infection was successful in all fawns, transmission of BVDV was documented in some, but not all calves, demonstrating that indirect contact may result in transmission of BVDV from deer to cattle (Ridpath et al., 2009).

During experimental cohabitation of pregnant white-tailed deer with two PI cattle in a 0.8 ha pen for 60 days, both species were observed to favor a common area in the pen enabling close interspecific contact (Passler et al., 2009). While direct interspecific contact was not noticed, deer were observed to use the feed trough shortly after the cattle. In that study, opportunity for direct and indirect BVDV transmission existed, and all does became infected with BVDV, resulting in the birth of PI fawns (Passler et al., 2009). In a follow-up study, one of the PI fawns was cohabitated with six pregnant white-tailed deer during the first trimester of gestation (Passler et al., 2010). The deer shared feed and water sources in an approximately 2 ha pen throughout gestation. All does became infected as result of exposure to the PI fawn and evidence of transplacental infection was detected, suggesting that PI white-tailed deer can readily transmit BVDV to in-contact animals (Passler et al., 2010). To date, quantification of BVDV that is shed by PI white-tailed deer has been reported only from one deer that was born to a doe infected with BVDV 2 PA131 (Passler et al., 2007). Viral titration of nasal swab and serum samples collected from this fawn (**Table 2**) demonstrated that PI deer can continuously shed BVDV in quantities that are similar to PI cattle.

**Table 2:** **Titration of BVDV in serum and nasal swabs from a persistently infected fawn**.

Day of sample collection	Serum virus isolation	Nasal swab virus isolation
8/25/06	6.2 × 10^5^ CCID_50_/ml	2 × 10^6^ CCID_50_/ml
9/25/06	6.2 × 10^5^ CCID_50_/ml	2 × 10^6^ CCID_50_/ml
10/23/06	6.2 × 10^5^ CCID_50_/ml	6.2 × 10^5^ CCID_50_/ml

*Adapted from Passler (2010)*.

## Maintenance of BVDV in Individual Hosts Or Host-Populations

Maintenance of BVDV in some populations of white-tailed deer may result from continual viral input from cattle when there is sufficient interspecific contact. While acutely infected deer may also play a role in the transmission and maintenance of BVDV in white-tailed deer populations, the greatest likelihood of independent maintenance would result from the presence of PI deer during the first trimester of gestation, which is influenced by the viability of PI deer, level of dispersion of PI deer, and gestational age at which a new generation of PI deer could be generated. In experimental infection studies, the viability of PI white-tailed deer fawns was markedly shorter than that of uninfected fawns, and most PI fawns did not survive beyond 1 month of age (Ridpath et al., 2008; Passler et al., 2009, 2010). However, survival to 5 and 10 months of age was reported for two other PI white-tailed deer in experimental infection studies (Passler et al., 2007, 2010). Kirchgessner et al. (2013) recently suggested that in New York, where the critical gestational period for generation of a new PI deer would be between mid-January to mid-February, based on an assumed critical gestational age of 50–67 days, PI fawns would have to survive for at least 8 months. The viability of PI white-tailed deer in free-ranging populations is currently unknown; however, the detection of PI animals in surveys of hunter-harvested white-tailed deer (Passler et al., 2008; Pogranichniy et al., 2008), suggests that some PI deer survive into adulthood.

The gestational age chosen for infection in studies that sought to generate PI white-tailed deer was based on extrapolation of the critical gestational age in cattle considering the shorter gestation length in deer, and was reported to be approximately 50–67 days (Passler et al., 2007; Ridpath et al., 2008). Reported gestational ages at time of infection of pregnant white-tailed deer that gave birth to PI fawns were 43, 42–49, and 41 days, respectively (Passler et al., 2007, 2010; Ridpath et al., 2007a). All PI fawns in studies by this research group were born to does infected between 27 and 51 days of gestation (**Table 3**), indicating that the critical gestational age in deer may be earlier than suggested by extrapolations from cattle. Therefore, environmental or behavioral factors that increase the amount of contact of pregnant white-tailed deer with PI livestock or deer before 50 days of gestation would increase the likelihood of BVDV maintenance in deer populations.

**Table 3:** **Gestational age and method of exposure in studies evaluating BVDV infection of white-tailed deer (Passler et al., 2007, 2009, 2010; Passler, 2010)**.

Fawn ID	Infection status	Date of birth	Method of exposure	Calculated age at exposure
GN	Persistently infected	8/25/2006	Intranasal	33
1	Seropositive	7/30/2007	PI cattle	56
2	Seropositive	7/30/2007	PI cattle	56
3	Seropositive	7/30/2007	PI cattle	56
4	Seropositive	7/30/2007	PI cattle	56
5	Persistently infected	8/4/2007	PI cattle	51
6	Persistently infected	8/15/2007	Intranasal	46
7	Persistently infected	8/21/2007	PI cattle	34
9	Seropositive	8/26/2007	PI cattle	29
10	Seropositive	8/26/2007	PI cattle	29
12	Persistently infected	8/28/2007	PI cattle	27
13	Seropositive	7/6/2008	PI deer	104
14	Seropositive	7/6/2008	PI deer	104
15	Seropositive	7/6/2008	PI deer	114
16	Seropositive	7/6/2008	PI deer	114
17	Seropositive	8/1/2008	PI deer	66
18	Seropositive	8/1/2008	PI deer	66
19	Seropositive	8/1/2008	PI deer	75
20	Seropositive	8/1/2008	PI deer	75
21	Persistently infected	8/13/2008	PI deer	41
22	Seropositive	8/16/2008	PI deer	63

*Calculation of the gestational age at the time of infection was based on a 200-day gestation length*.

A recent study conducted in New York analyzed whether areas with high BVDV seroprevalence rates in white-tailed deer were associated with greater rates of BVDV antigen-positive cattle and camelid herds, and identified three unique scenarios of BVDV epidemiology (Kirchgessner et al., 2013). In central New York, focal areas of elevated prevalence rates of BVDV antigen in livestock and BVDV antibodies in white-tailed deer were identified, indicating that cattle, camelids, and deer served together as a host community for BVDV. In contrast, in western New York, the greater rate of BVDV antigen prevalence in livestock was not associated with increased rates of seroprevalence in white-tailed deer. Interestingly, the western part of New York reportedly contained the greatest deer densities, indicating that the rate of BVDV transmission between cattle and deer is not dependent on deer densities (Kirchgessner et al., 2013), which was previously reported for white-tailed deer in Mexico (Cantu et al., 2008). In northern New York State, an area with low deer density, a cluster of high BVDV seroprevalence among white-tailed deer was detected. In that area of the state, the BVDV antigen prevalence in livestock was low, suggesting that BVDV was independently maintained in the white-tailed deer population. The authors suggested that rather than being a function of deer density, BVDV transmission among white-tailed deer is associated with deer behavior and migration patterns, including congregation in winter yards (Kirchgessner et al., 2013).

Deer wintering behavior in northern regions likely contributes to increased BVDV prevalence. Deer wintering areas are frequently characterized by very high densities of deer that are generally restricted to trail systems due to extreme snow depths. High contact rates between deer on these trail systems and at common food sources (Schmitt et al., 1997) could increase transmission rates, particularly if PI animals were present. Because deer wintering areas contain deer that may migrate from more than 30 miles away (Verme, 1973), rather than just containing deer that reside in close proximity to the wintering area, the potential exists in these habitats to expose deer populations to BVDV that reside in cattle-free areas. In contrast, more sedentary deer populations that are found where snow depths are not restrictive during winter may not have exposure rates as great, nor the potential for deer residing in cattle-free areas to be exposed. Additionally, the timing of congregation in wintering areas would likely increase the prevalence of PI deer. Because deer congregate in these areas during January–March (Ozoga and Gysel, 1972) and most pregnant does will be approaching the end of their first trimester in mid-January (Verme, 1977), the probability of producing PI fawns would be significantly elevated if PI animals were present. Finally, it is very common for supplemental food sources to be available to deer when in wintering areas. Local human residents often feed wintering deer to reduce overwinter mortality (Milner et al., 2014), and it is not uncommon for groups of 50–100 deer to be found at individual feed sites at the same time. The close proximity of these animals at, and sharing of, common food sources would significantly elevate exposure to BVDV if the virus was present in the wintering population. This scenario has led to high transmission rates of bovine tuberculosis in wintering populations of white-tailed deer in Michigan (Schmitt et al., 1997; Miller et al., 2003). More southerly deer populations would not experience this period of elevated exposure.

## Contact of White-Tailed Deer with Cattle

Factors that affect the transmission of BVDV in cattle populations include the duration of the infectious period, the presence of susceptible hosts that lack immunity necessary to prevent infection, infectiousness of the virus strain, and the number of adequate contacts between BVDV-infected and susceptible animals. The same factors likely also apply to maintenance of BVDV in populations of white-tailed deer and determine whether white-tailed deer can serve as a BVDV reservoir and cause spill-back infection to cattle. While shedding and transmission of BVDV was demonstrated in white-tailed deer, there is currently sparse information on how passage of BVDV through deer affects the infectivity of the virus for cattle and whether sufficient contact occurs between acutely infected or PI white-tailed deer and susceptible cattle. The occurrence of ‘sufficient contacts’ is key to the discussion of BVDV transmission from deer to cattle, and theoretically, both direct and indirect routes can result in transmission of BVDV between deer to cattle. A recent study that evaluated the co-occurrence of pathogens with either direct or indirect transmission route in cattle herds with or without exposure to elk determined that only indirectly transmitted pathogens co-occurred in both species (Pruvot et al., 2014).

There are many anecdotal reports of close contact between white-tailed deer and cattle in pastures and at feed and water sources that may promote direct interspecific transmission of BVDV. In a survey conducted by the United States Department of Agriculture, 49.3% of dairy operations reported deer or other members of the deer family had physical contact with dairy cattle or their feed, minerals, or water supply (United States Department of Agriculture, Animal Plant Health Inspections Service, and Veterinary Services, 2011). On operations on which contact of cattle with cervids occurred, 90.8% of farmers reported that cattle could possibly or sometimes have face-to-face contact with deer. In a similar survey of beef cattle producers, 72.6% of operations reported that cattle had physical contact with wild cervids (United States Department of Agriculture, Animal Plant Health Inspections Service, and Veterinary Services, 2011). A study in southwestern Manitoba reported that nearly 100% of cattle producers had observed the presence of white-tailed deer on their farms (Brook et al., 2013). Of the interviewed farmers, 11 and 47% had observed direct or indirect contact, respectively, between white-tailed deer and cattle (Brook et al., 2013). In contrast to results of farmer surveys, sufficient direct contact of cattle and white-tailed deer was rarely reported in studies using visual observations or Global Positioning System (GPS) collars to study the spatial distribution of both species. In a 2-year-study in Michigan in which the number of contacts of white-tailed deer with other species were visually observed, only one direct contact and 273 indirect contacts between deer and cattle were recorded (Hill, 2005). Similarly, close contact of cattle and white-tailed deer was rare in a study in Texas, and deer tended to be displaced by cattle approaching at a distance of within 46 ± 5 m (Cooper et al., 2008). While cattle generally tolerate the presence of deer, deer tended to avoid cattle at distances lower than 50 yards (Krämer, 1973). The social relationships of cattle and deer are controversially discussed in the published literature (Krämer, 1973), and contact of both species is influenced by various factors including habitat type, season, presence and type of supplemental feed for cattle, and presence of barrier fencing at feed storage sites (Brook, 2010; Brook et al., 2013; Lavelle et al., 2015).

Indirect routes are likely to be more important for BVDV transmission than direct contact between deer and cattle; thus, virus survivability and distance from infected animals to susceptible animals are important factors contributing to indirect transmission of BVDV from deer to cattle. Since BVDV is an enveloped virus, the virus is unstable at low or high pH (Houe, 1995). In addition, temperature impacts the survivability of BVDV, which remained infective for greater than 6 weeks in manure slurry stored at 5°C but less than 2 weeks at 20°C (Botner and Belsham, 2012). Enhanced BVDV survivability at colder temperatures in combination with a greater potential for wild cervids and livestock to make indirect contact at common feed sources during winter months when forages are scarce (Brook et al., 2013), suggest a greater risk of indirect transmission during colder winter months. Food and water are aggregation points for cattle and deer, and BVDV can be transmitted horizontally via oral and nasopharyngeal secretions. Since oral and nasopharyngeal secretions contain mucus, survivability and infectivity were compared in fomites contaminated with BVDV in aqueous or mucus solution, and BVDV appeared to survive for longer periods of time on most fomites in the presence of mucus (Stevens et al., 2009). In addition, BVDV could be recovered at significantly greater levels and for longer periods of time in water in the presence of mucus than without mucus; however, this research was performed under laboratory conditions, and survivability could be potentially enhanced or reduced under different environmental and climatic conditions (Stevens et al., 2009). BVDV survives for up to 60 days in tissues of PI cattle, and the potential for BVDV transmission from carcasses of white-tailed deer to susceptible cattle was recently evaluated (Passler et al., 2012a).

Insects, especially those requiring blood meals such as mosquitoes and tabanid flies may also have the potential to serve as a source of indirect BVDV transmission (Tarry et al., 1991). Horse and deer flies are tabanid flies, of which there are an estimated 4,300 different species worldwide. The female flies are aggressive blood feeders, and are capable of feeding on many different types of mammals. Horse flies were able to transmit BVDV to susceptible cattle after feeding on a PI steer (Tarry et al., 1991); however, the ability to transmit from PI deer to cattle, while conceptually possible, has not been demonstrated. Many species of mosquitos lack mammalian host specificity, and thus could also be a potential source of mechanical transmission between wildlife and livestock. Although insects could serve as an indirect route of transmission, no epidemiologic data are available to estimate the risk of arthropods as transmitters of BVDV infection from deer to cattle or vice versa.

## Summary

Bovine viral diarrhea virus is a ubiquitous pathogen capable of infecting more than one host species. A key issue in the design of BVDV control measures is to determine if heterologous hosts constitute an infection reservoir. In order to serve as an infection reservoir, four key requirements must be met including: (1) susceptibility to BVDV, (2) BVDV maintenance, (3) BVDV transmission, and (4) sufficient contact that allow spillback infections. With respect to susceptibility to BVDV infection, experimental infection studies which are corroborated by epidemiologic investigations provide strong evidence that BVDV infection occur in white-tailed deer, including transplacental infections and birth of PI offspring. Furthermore, BVDV can be maintained in white-tailed deer populations as strongly evidenced by epidemiologic data indicating high seroprevalence rates. Persistently infected deer are described, and these deer are capable of shedding BVDV at levels consistent with PI cattle. Some PI deer were described through epidemiologic investigations to survive into adulthood providing strong evidence that deer can be important sources of BVDV for susceptible animals. Finally, evidence that PI deer and naïve cattle make sufficient contact to result in spillback infections to cattle is weak. While data exist that indicate deer and cattle make direct contact and that potential indirect contact exists at food and water aggregation points, the low prevalence of PI deer along and scarcity of sufficient contacts between PI deer and naïve cattle suggest a low risk for white-tailed deer as an important reservoir of BVDV in most environments. BVDV infections should be considered a threat to the health and reproductive success of deer, but the greatest risk for BVDV infection in cattle likely resides in PI cattle.

## Author Contributions

TP, SD, and PW contributed to the preparation, review, and revision of the manuscript.

## Conflict of Interest Statement

The authors declare that the research was conducted in the absence of any commercial or financial relationships that could be construed as a potential conflict of interest.
